# Servants and Medical Men

**Published:** 1896-12-12

**Authors:** 


### SERVANTS AND MEDICAL MEN.

We have received the following note, which is of considerable interest to medical men: A somewhat interesting case in which a member of the Society was concerned was dealt with lately by the Council of the London and Counties Medical Protection Society, Limited. The facts are shortly these: A doctor was called to a patient's house, and he was asked by his employer to see and prescribe for her maid-servant and to report to the employer upon the girl's state of health. In consequence of the doctor's report, the servant was dismissed. She commenced an action for slander against the doctor, who finally, upon the advice of the Council of the Society, settled the matter by paying her a solatium, the costs of the case being borne by the Society. The solicitors to the Council advised that a medical man paid by his employer to attend upon the servant of the employer (the servant not objecting to being attended by the doctor) might divulge to his employer the result of his attendance, that being a privileged communication; but if the report is made in the presence of, or to any other person than the employer, as happened in the above instance, the report is not privileged, and the matter may become actionable. The Council expressed a distinct opinion that a doctor consulted by an employer in reference to the health of a servant should obtain the written consent of the servant before prescribing or divulging the result of his examination to the employer in such cases where the servant might be likely to object to his doing so.

*** We have no doubt that the legal aspect of the case is accurately expressed in the opinion of the solicitor, as given above; but, unless care is taken to obtain written consent, as suggested by the Council, a question of fact will always be open to dispute, viz., whether the servant understood the condition on which the consultation took place. Hence the recommendation of the Council.—Ed. *T. H.*

